# Sing the Genome Electric: Excited Cells Adjust Their Splicing

**DOI:** 10.1371/journal.pbio.0050055

**Published:** 2007-02-13

**Authors:** Manuel Ares

## Abstract

Neuronal depolarization regulates the alternative splicing of NMDA receptor subunits, providing molecular insight into how experience alters gene expression.

Those of us who study alternative splicing like to think it is the driving force behind the rapid evolution of human nature. Of course, we could be wrong, but in complexity and capability of the human species without a corresponding increase in gene number? Humans have an estimated 20,000–25,000 protein-coding genes, compared to about 19,200 for worms. Are we simply very smart worms? Our complexity is most evident (to us) in that most special of special human features, the brain. How did only 5 million years of evolution squeeze so much out of the primate genome so quickly?

Perhaps it is not so much the written poem, but the reading that matters. While it is true that RNA polymerase controls what part of the genome to read and when, it is the spliceosome and its splicing factors—the machinery that removes noncoding information from nascent gene transcripts to make messenger RNA (mRNA)—that provide the interpretive pace, phraseology, emphasis, and intonation to the reading. The splicing machinery, through the process of alternative splicing, can produce different mRNAs and hence different proteins from the same gene, depending on the biological circumstances. Human genes produce on average three distinctly spliced alternative mRNAs rather than only one, and some genes have the potential to produce thousands of distinct mRNAs through alternative splicing. The view that alternative splicing adds the genetic complexity that makes us human may help us feel better about sharing most of our genes with less psychologically complex organisms. But is it true? Perhaps there is more to it than our own self-absorption. In this issue of PLoS Biology, An and Grabowski [1] and Lee et al. [2] connect the electrical activity of neurons with complex and coordinated regulation of gene expression at the level of alternative splicing.

## How Is Alternative Splicing Regulated?

Alternative splicing regulation remains surprisingly mysterious. For splicing to create functional mRNA, introns (the part of the pre-messenger RNA [pre-mRNA] to be removed) must be distinguished from exons (the part of the pre-mRNA to be retained in the mRNA). To achieve this, the splice sites—the places in the pre-mRNA where the spliceosome must cut and religate the transcript—must be paired with high precision, to ensure that no exons are skipped. Alternative splicing allows these precise pairings to be subject to genetic regulatory control, in order to change the content of the mRNA and the function of the encoded protein product. But how? It seems clear so far that the decision about which splice site will be joined to which has much to do with where the spliceosomal subunits (called small nuclear ribonucleoproteins [snRNPs]) bind to the pre-mRNA [3]. These subunits bind to the pre-mRNA and assemble with each other through an orderly process to make the active spliceosome. This assembly process is both promoted and inhibited by splicing factors that bind to the adjacent pre-mRNA and influence which splice sites are selected. How positive and negative influences are actually manifested on the snRNPs at the biochemical level is far less clear, but it's possible that stabilization of protein–protein interactions may cooperatively increase the affinity of snRNPs for an RNA region, or that prior binding of a splicing factor may simply occlude snRNP binding [3]. There are abundant hints that things are not so simple, and that later steps of spliceosome assembly and function are subject to regulation as well (e.g., [4–6]). But the emphasis on regulation at the point of spliceosome assembly has naturally made the splicing factors that bind to pre-mRNA a favorite subject of study.

## The Yin and Yang of Splicing Factor Activity

There are many splicing factors to learn about [7]. The two most intensively studied, ASF/SF2 and heterogeneous nuclear ribonucleoprotein (hnRNP) A1, have antagonistic roles in determining whether an exon is included in the mature mRNA or skipped. ASF/SF2 belongs to the SR family of proteins, so named because of their serine-arginine repeat domains. In general, SR proteins like ASF/SF2 promote exon inclusion by binding to short sequences in the RNA called exonic splicing enhancers and encouraging spliceosome assembly at nearby splice sites. On the other side are the hnRNP proteins such as hnRNPA1 that bind to other short sequences called exonic splicing silencers and interfere with correct spliceosome assembly at nearby splice sites, leading to skipping of the exon [3]. In many exons, even for those “constitutive exons” not apparently regulated by alternative splicing, there exists a dynamic tension between exon inclusion and exon skipping whereby both SR proteins and hnRNP proteins contribute to recognition of the correct splice sites, presumably by activating correct sites while repressing use of nearby incorrect sites. If an exon is altered so that it loses some affinity for an SR protein or gains affinity for hnRNPA1, it may be skipped [3]. If the balance is changed by overexpression of one relative to the other, exons respond [8]. So it seems that each exon makes a hard bargain with the positive and negative splicing factors such that its accurate and efficient inclusion is ensured. This also means that the overall balance of positive and negative splicing factors in the cell must be tightly controlled, or else nearly every exon will struggle to be properly included.

In addition to their roles in the basal splicing of constitutive exons, SR protein and hnRNP protein splicing factors also mediate programmed changes in splicing at alternatively spliced exons. Not surprisingly, the bargain that alternative exons make with the two antagonistic sets of basal splicing factors must be more nuanced, in order to respond to the added influence of a splicing factor whose own activity is regulated by some external signal. Evidence for these more complex demands comes from observations that the splice sites bordering alternative exons tend to be weaker, and the intron regions near alternative exons are more highly conserved [9–13]. Like the basal SR protein and hnRNP protein splicing factors, more specialized alternative splicing factors also bind the pre-mRNA at special enhancers or silencers in either the exon (exonic splicing enhancers and exonic splicing silencers) or the intron (intronic splicing enhancers and intronic splicing silencers). An example of such a factor is the neural-specific Nova-2 splicing factor, found only in certain neural cells where it regulates splicing of synaptic protein mRNAs [14]. Current areas of intensive research in alternative splicing are (a) to determine how signals are transmitted to the splicing apparatus and (b) to identify as many responding exons as possible, in order to understand the logic and combinatorial networks of alternative splicing regulation.

## Alternative Splicing and Neurons

But does alternative splicing contribute to establishing the cellular and molecular groundwork of learning and memory? It is notably widespread in the nervous system [15,16]. Alternative splicing seems an attractive means to create self-perpetuating, metastable, long-term changes in gene expression patterns, as would seem necessary for long-term cellular memory. The time scale over which splicing changes can be created is slow compared to protein phosphorylation, for example, because pre-existing mRNAs must decay and newly spliced RNAs take their place. Positive feedback regulation of splicing is known to be capable of generating an epigenetic state, such as in the determination of female identity in flies by the splicing factor Sxl [17]. Thus, as a form of regulated protein modification, alternative splicing has dynamics and persistence consistent with the time scale of learning and long-term memory. In addition, excitation of neural cells in culture leads to changes in splicing that could affect the number and strength of synapses in the responding cell through altered function of neurotransmitter receptors and other ion channels [15,16]. One splicing change that has attracted attention because of its potential impact on the responsiveness of neurons is that involving the C1 exon of the NMDA receptor, a calcium-permeable channel that opens in response to the neurotransmitters glutamate and glycine and triggers long-term changes in synapses [18,19]. Inclusion or skipping of the C1 exon, and the consequential presence or absence of the C1-encoded peptide sequence in the NMDA NR1 subunit, has multiple impacts on the function of the receptor protein [20–25].

**Figure 1:**
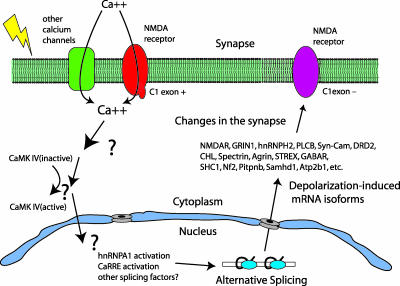
Alternative Splicing in Response to Depolarization in Neurons Depolarization of the cell (yellow lightning bolt) leads to an influx of calcium. This influx activates a cascade leading to the activation of CaMK IV. The signal is transmitted by unknown means to the splicing apparatus. One consequence is an increase in the amount and activity of hnRNPA1, which represses inclusion of the NMDA NR1 exon 21 (C1 exon) through a compound hnRNPA1-responsive element. This repression also requires multiple CaRREs in the same exon. A wide variety of other splicing events are also altered, many of which occur in proteins that, like the NMDA receptor, are restructured by alternative splicing and also influence the function of the synapse.

Both An and Grabowski [1] and Lee et al. [2] set about to understand the depolarization-regulated splicing silencers within and near the C1 exon that lead to its increased skipping in response to excitation. Cultures of cortical neurons or differentiated P19 cells can be treated with potassium chloride (KCl) to depolarize them, simulating the excited state (Figure 1). Under these conditions, splicing changes so that the C1 exon is skipped, and the pool of NMDA receptor mRNA shifts in its composition. The change is reversible upon washout of KCl. These changes can be followed using splicing reporters, and the sequences responsible for depolarization-dependent repression of the C1 exon can be dissected by mutagenesis. An and Grabowski identified multiple hnRNPA1 binding sites within the exon that are together required to mediate the depolarization-dependent repression of the C1 exon. They surgically introduced this compound element into a different exon that is not responsive to depolarization and successfully transplanted the depolarization-dependent splicing repression. Using the C1 exon RNA sequence as an affinity matrix to capture proteins that might bind to their silencer sequence, An and Grabowski showed that depolarization stimulates binding of hnRNPA1 to the C1 exon. Thus, repression of the C1 exon was achieved in part by activation of hnRNPA1 in excited cells (Figure 1). A search for new depolarization-responsive exons revealed many new splicing events, a number of which are associated with hnRNPA1 binding sites in genes with synaptic functions.

Lee et. al. [2] found different elements within the C1 exon that do not overlap the hnRNPA1 recognition sites. In some of their experiments, they created a signaling environment very like that found in the depolarized cells by expressing a constitutively activated form of calmodulin-dependent protein kinase IV (CaMK IV) in place of KCl treatment [2]. This situation mimics the excited cell, in which depolarization-dependent calcium influx would normally activate the kinase (Figure 1). Lee et al.'s mutagenesis study revealed a new CaMK IV–responsive RNA element (CaRRE)–like sequence element similar to that previously reported by this group [26]. This new element, as well the original CaRRE, can be transplanted to an exon that is not regulated by depolarization, and can mediate repression of this new exon under depolarization conditions. Lee et al.'s detailed mutagenesis study provided a clear sequence profile of the CaRRE sequence elements, permitting the researchers to search for other exons in the genome that might be regulated by depolarization. Significantly, 16 of 27 of the newly identified CaRRE-containing exons are responsive to depolarization in differentiated P19 cells, whereas 16 of 17 exons deemed not to have a CaRRE were unresponsive. Thus, the ability to gain strongly predictive information from a comprehensive mutagenesis effort has revealed a number of new splicing events regulated by depolarization.

## Where Next?

To be sure, there are a number of caveats and issues for the future. For example, how well does chronic depolarization by KCl treatment of cell cultures mimic the excited state? Are these responses related to homeostasis of a responsive state, or are they truly connected to the molecular changes that record the experience of a single neuron within a network? Current technology would be sorely stretched to measure global changes in splicing in such individual cells, but this is clearly an important next question. In addition, the proteins that bind to the CaRRE are unknown, as are the factors that connect calcium signaling to the activation of hnRNPA1 in these cells. In fact, for very few, if any, instances of regulated alternative splicing is there a clear path from the signaling event to the splicing machinery [27], a deafening silence in the general conversation on signal transduction pathways. In addition, more studies will be required to determine whether these splicing changes contribute in important ways to the modification of cellular behaviors that characterize the phenomena of learning and memory—and to perhaps shed light on the genome-regulating mechanisms that underlie our cognitive complexity. But together, the two papers by An and Grabowski, and Lee et al. provide exciting new insights into how neurons alter their gene expression in response to their experience.

## Abbreviations

CaMK IV: calmodulin-dependent protein kinase IV
CaRRE: calmodulin-dependent protein kinase IV–responsive RNA element
hnRNP: heterogeneous nuclear ribonucleoprotein
KCl: potassium chloride
mRNA: messenger RNA
pre-mRNA: pre-messenger RNA
snRNP: small nuclear ribonucleoprotein
